# Photonic eigenmodes and transmittance of finite-length 1D cholesteric liquid crystal resonators

**DOI:** 10.1038/s41598-023-43912-2

**Published:** 2023-10-06

**Authors:** Jaka Zaplotnik, Urban Mur, Deepshika Malkar, Amid Ranjkesh, Igor Muševič, Miha Ravnik

**Affiliations:** 1 Faculty of Mathematics and Physics, University of Ljubljana, 1000 Ljubljana, Slovenia; 2 Department of Condensed Matter Physics, Jožef Stefan Institute, 1000 Ljubljana, Slovenia

**Keywords:** Optics and photonics, Liquid crystals, Microresonators, Photonic crystals, Micro-optics, Liquid crystals

## Abstract

Cholesteric liquid crystals exhibit a periodic helical structure that partially reflects light with wavelengths comparable to the period of the structure, thus performing as a one-dimensional photonic crystal. Here, we demonstrate a combined experimental and numerical study of light transmittance spectra of finite-length helical structure of cholesteric liquid crystals, as affected by the main system and material parameters, as well as the corresponding eigenmodes and frequency eigenspectra with their *Q*-factors. Specifically, we have measured and simulated transmittance spectra of samples with different thicknesses, birefringences and for various incident light polarisation configurations as well as quantified the role of refractive index dispersion and the divergence of the incident light beam on transmittance spectra. We identify the relation between transmittance spectra and the eigenfrequencies of the photonic eigenmodes. Furthermore, we present and visualize the geometry of these eigenmodes and corresponding *Q*-factors. More generally, this work systematically studies the properties of light propagation in a one-dimensional helical cholesteric liquid crystal birefringent profile, which is known to be of interest for the design of micro-lasers and other soft matter photonic devices.

## Introduction

The major optical property of cholesteric liquid crystals (CLCs) is selective light reflection^1–8^ due to the periodicity of their spontaneously formed helical birefringent structure. For the incidence of light along the helical axis of a CLC, a birefringent nature of LC molecules in combination with rotating nematic director results in a photonic band gap for the wavelengths, comparable to pitch *p*, the distance along the helical axis that corresponds to a rotation of the director of $360^{\circ }$. Such band gap exists only for a circularly polarised light with the same handedness as the helix, which is strongly reflected. Due to selective light reflection, CLCs are 1D photonic band gap materials and allow for the implementation of tunable optical filters^9–11^ and isolators^12^, light shutters^13^, diffractive optical devices^5^ and band edge lasers^14–19^.

The spatially modulated birefringent structure of CLCs performs as an optical resonator, where the resonances occur due to the Bragg reflection^20^. Lasing in such a one-dimensional periodic structure is achieved when the optical gain material—typically, a fluorescent dye—with the emission spectrum overlapping with one of the edges of the band gap is added to the system to amplify the light. When the dye molecules are illuminated by short pulses, they emit photons, which undergo the process of stimulated emission. Eventually, lasing at the frequency determined by the dye emission spectrum and periodic structure of the resonator occurs. CLC lasers are attractive for optical applications due to the ease of fabrication (i.e. the periodic structure is essentially self-assembled) and tunability^21^: the pitch and consequently the laser emission wavelength can be tuned via temperature^22^, mechanical strain^23,24^, external electric fields^25^, phototuning^26^, by optically inducing the material flow^27^ or changing the material composition^28^. In addition, also the emission direction can be tuned^29^ and defect mode lasing can be realised^30,31^.

1D CLC resonators have been extensively explored in the past, both experimentally and with modelling or theory. Theoretically, 1D, 2D or 3D periodic materials are studied in terms of band structures and band gaps, which are normally calculated for infinite materials without boundaries^32^. In practice, resonators, including 1D CLC resonators, have finite size (i.e. finite number of unit cells which are repeated periodically within the boundaries of the material), which significantly affects the photonic response. Transmittance and reflection coefficients for finite size CLC layer have been determined numerically by matrix methods^33^, and finite element method^34^ as well as the lasing thresholds^35^. Similarly, numerical analyses of the effects of dielectric boundaries (substrate) on light localization, light coupling into different modes of the cavity, the effects of dielectric boundaries (substrate) on light localization and dependence of light localization on the CLC layer thickness have been done^36,37^. In Ref.^38^ it is shown that the rotatory power of cholesterics consists of two parts: one of them is related to the diffraction-induced circular dichroism whereas the other one originates from the tails of the Mauguin rotation of the plane of light polarisation, in Ref.^39,40^ the density of states and in Ref.^41^ spectral and polarisation characteristics of the light passing through a CLC are analyzed. There has been less research done on the localized modes, which are relevant for lasing. In Ref.^42^ localized edge modes have been theoretically described and coupling into such modes has been demonstrated numerically for normal^36^ and oblique incidence^43^.

In this work, we demonstrate a combined experimental and numerical study of light transmittance spectra through finite-length helical structure of cholesteric liquid crystals, and the corresponding eigenmodes and frequency eigenspectra. Specifically, we have measured the transmittance spectra of samples with different thicknesses, birefringences and for various incident light polarisation configurations and compared them with numerical spectra obtained by Finite-Difference Time-Domain (FDTD) method. We quantify the role of refractive index dispersion and of the divergence of the incident light beam on the transmittance spectra. Next, we numerically calculate, using the Finite-Difference Frequency-Domain (FDFD) method, the full spectrum of the (passive) resonant eigenmodes and explore their belonging quality factors. We use the numerical results to identify the relation between transmittance spectra and the eigenfrequencies of the photonic eigenmodes. Furthermore, we present and visualize the geometry of these eigenmodes and calculate the corresponding *Q*-factors. Compared to previous work on standing optical edge modes, solving Maxwell’s equations in the full vector eigenproblem form gives us the exact solutions for the electric field within the cavity. Therefore, we gain an in-depth insight into the coupling between light and liquid crystal gain medium, which is essential information when orientable dies are used in lasers^44,45^. More generally, this work is aimed to contribute to the development of soft matter based photonic platforms for advanced manipulation and control of the flow of light.

## Results

**Figure 1:**
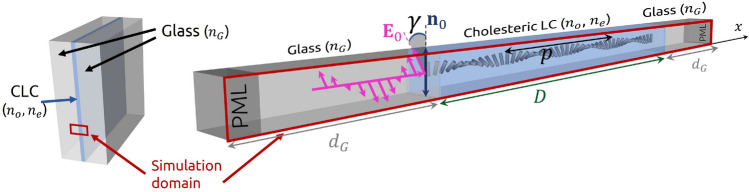
Schematic of the liquid crystal geometry. CLC sample with ordinary refractive index $n_o$, extraordinary refractive index $n_e$ and pitch *p* is confined between two thick glass plates with refractive index $n_G$. 1D simulation domain, marked in red, consists of a CLC layer with thickness *D* and effectively infinite glass layers on each side, which is achieved by use of PML (perfectly matched layers). Linearly polarised light with polarisation ${\textbf{E}}_0$ enters the CLC at an angle $\gamma ({\textbf{n}}_0,{\textbf{E}}_0)$, relative to the nematic director orientation at the incident surface ${\textbf{n}}_0$. In all simulations, the light source (the incoming light shown in pink here) lies within one of the glass plates confining the CLC.

Photonic eigenmodes and transmission spectra are explored in finite-length cholesteric liquid crystal resonators are using complementary numerical FDTD and FDFD modelling and experiments, as described in Methods. The cholesteric liquid crystal sample of thickness *D* with ordinary $n_o$ and extraordinary refractive index $n_e$ is confined between two equal $d_G$ thick glass plates with refractive index $n_G=1.5$, as schematically shown in Fig. 1. The configuration of the director in the CLC sample with helical axis along *x*-axis can be written as ${\textbf{n}}(x)=(0,\ \cos (2\pi (x-d_G)/p),\ \sin (2\pi (x-d_G)/p))$, where *p* is the cholesteric pitch. The given director field corresponds to the following dielectric tensor

1 $$\begin{aligned} \underline{\varepsilon }_{ij}=n_o^2\delta _{ij}+(n_e^2-n_o^2)n_in_j, \end{aligned}$$

where $i,j\in \{x,y,z\}$, $\delta _{ij}$ is the Kronecker delta, and $n_i$ are components of the director ${\textbf{n}}(x)$.

### CLC transmittance spectra

Light with wavelengths $n_op< \lambda < n_ep$ propagating along the helical axis of a cholesteric liquid crystal is partially reflected. The circular polarisation component with the same handedness as the liquid crystal structure is reflected, while the opposite polarisation is transmitted. First, we present transmittance spectra obtained by experimental measurements and FDTD numerical modelling, depending on main system parameters, including refractive indices ($n_o$ and $n_e$), pitch length (*p*), and thickness (*D*), as well as the polarisation of the incident light. We also consider the dispersion relations of refractive indices and experimentally relevant modulation of the incident light, beyond the usual theoretical assumption of plane waves.

#### Role of sample thickness (for fixed pitch)

**Figure 2:**
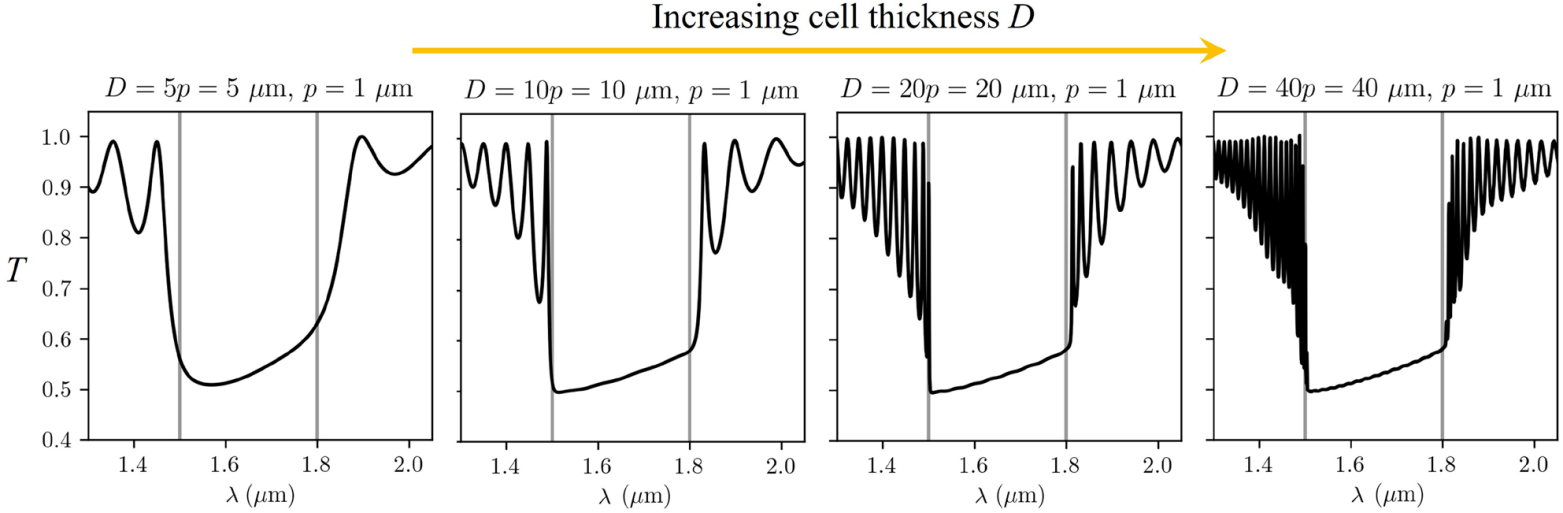
Simulated transmittance spectra in differently thick cholesteric liquid crystal cells with refractive indices $n_o=1.5$, $n_e=1.8$ and pitch $p={1}\,\upmu \hbox {m}$. The angle between the electric field’s polarisation and the director field at the incident surface is $\gamma ({\textbf{n}}_0, {\textbf{E}}_0)=0^\circ$ here. In all numerical simulations, only glass–CLC–glass transmission is used in calcualation, not taking into account possible reflections at the external glass-air interface, possibly relevant in experiments.

Figure 2 demonstrates the role of the finite size of the cholesteric helical pattern, where we vary the thickness of the cholesteric cell but keep the cholesteric pitch constant, essentially varying the number of Bragg layers of the cholesteric resonator. We observe that with increasing sample thickness, the frequency range at which light is reflected does not change significantly, but the transmittance spectrum does. Approximately half of linearly polarised light with wavelengths $n_op< \lambda < n_ep$ is reflected by already relatively thin structures; for example, five pitch lengths thick ($D=5p$) sample with $\Delta n=n_e-n_o=0.3$ give transmittance $T\lesssim 0.6$ for the band gap wavelengths. The spectra of light transmitted through thicker samples contain more oscillations outside the photonic band gap and appear sharper at the band edge.

#### Experimentally measured spectra of different materials

**Figure 3:**
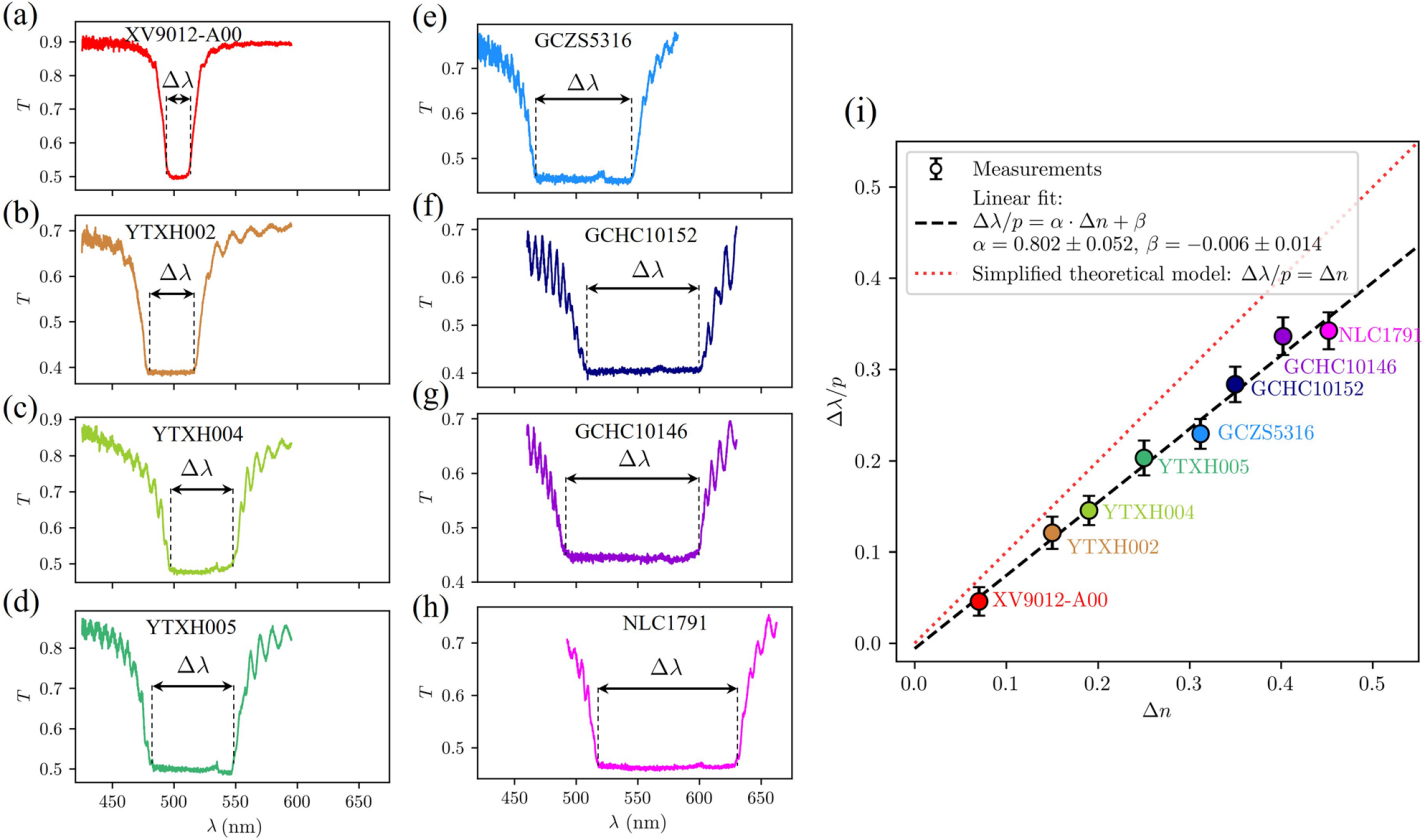
(**a**–**h**) Experimentally measured unpolarised light transmittance spectra for eight cholesteric liquid crystal samples in $8\,\upmu\hbox{m}$ thick cells with different birefringences. Note, that experimental measurements include reflections on glass-air interfaces of the CLC cells. (**i**) Corresponding dependence of the normalized bandwidth $\Delta \lambda /p$ on the nematic birefringence $\Delta n$. Cholesteric pitch lengths and other material properties are listed in Table 1.

**Figure 4:**
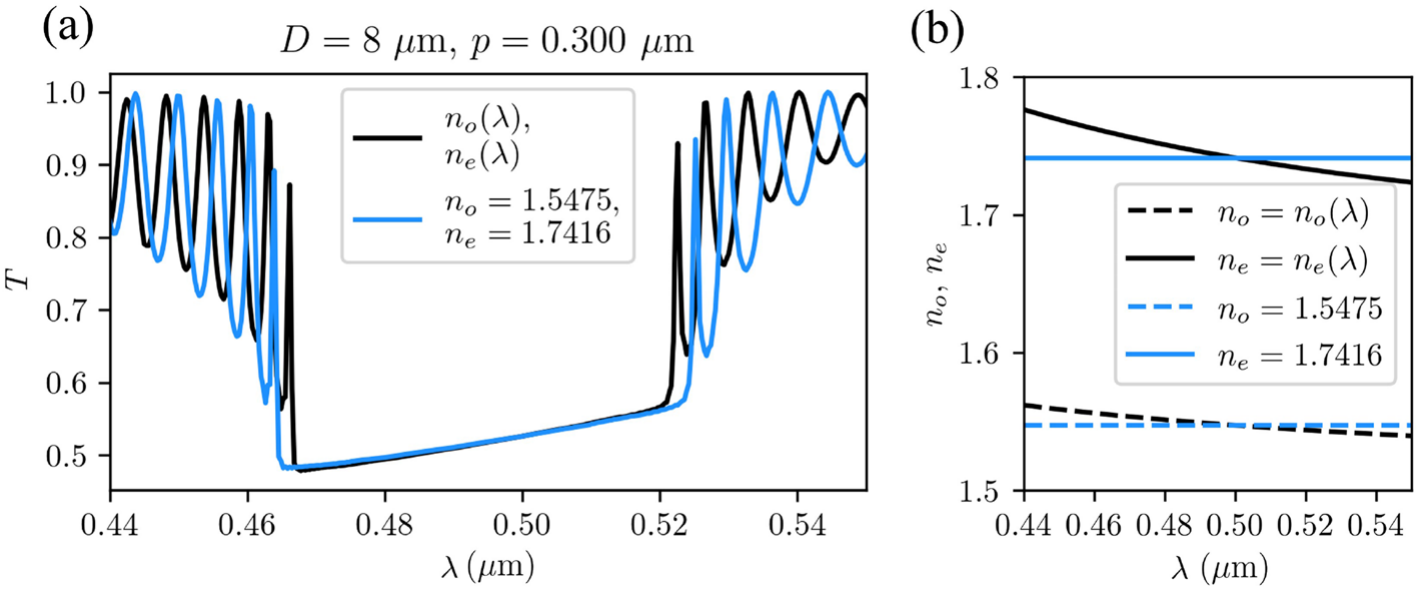
Effect of refractive indices dispersion ($n_o(\lambda )$, $n_e(\lambda )$) on transmittance spectrum. (**a**) The blue line shows the calculated spectrum of transmitted light for wavelength-independent refractive indices ($n_o=1.5475$, $n_e=1.7416$), and in black, the spectrum of the transmitted light for wavelength-dependent refractive indices with dispersion relations $n_o(\lambda /\upmu \text {m})= 1.5139 + 0.0052\ \lambda ^{-2} + 0.0008\ \lambda ^{-4}$, and $n_e(\lambda /\upmu \text {m})= 1.6708 + 0.0081\ \lambda ^{-2} + 0.0024\ \lambda ^{-4}$, are shown. (**b**) Corresponding dispersion relations.

The wavelength range at which light is reflected on a CLC is most affected by the refractive indices and the pitch length. A systematic experimental study has been performed where we have measured the spectra of transmitted unpolarised light $T(\lambda )$ for eight different liquid crystals with different nematic birefringences $\Delta n= n_e-n_o$ and similar pitch lengths *p* (see Table 1), which are shown in Fig. 3. The bandwidths $\Delta \lambda =\lambda _2-\lambda _1$ measured between the edges of the flat bottom of the spectrum, as shown in the Fig. 3, linearly depend on birefringence $\Delta n$. Our numerical simulations and also other theoretical approaches^3, 34^ predict the edges of the photonic band gap at wavelengths $\lambda _1=n_o p$ and $\lambda _2=n_ep$, and thereby a linear relation $\Delta \lambda /p=\Delta n$, while in this case, the linear relation $\Delta \lambda /p \approx 0.8 \Delta n$ holds. The difference could be a consequence of a combination of several reasons: (i) The refractive indices of nematic liquid crystals change after a chiral dopant is added, and it is challenging to measure them after doping, but typically, the birefringence decreases after adding the dopant, and (ii) the refractive indices depend on the wavelength of light. In the simplified numerical model which gives $\Delta \lambda /p=\Delta n$, the dispersion relations of refractive indices is neglected. The impact of the wavelength dependence of the refractive indices on transmittance spectra is shown in Fig. 4.

**Table 1:** Properties of liquid crystals used in experiments: clearing temperature $T^*$, birefringence $\Delta n$ as determined in the nematic phase prior to the addition of chiral dopants; and measured quantities: chiral dopant concentration *C*, pitch length *p*, band edges $\lambda _1$, $\lambda _2$, and bandwidth $\Delta \lambda =\lambda _2-\lambda _1$.

LC sample	$T^*$ ($^\circ$C)	$\Delta n$	C (wt%)	*p* (nm)	$\lambda _1$ (nm)	$\lambda _2$ (nm)	$\Delta \lambda$ (nm)
XV9012-A00$^{(i)}$	84	0.07	1.125	335 ± 5	496	511.5	15.4
YTXH002$^{(ii)}$	90	0.15	1.114	318 ± 5	479.2	517.8	38.5
YTXH004$^{(iii)}$	90	0.19	1.076	329 ± 2	498.9	545.8	47.9
YTXH005$^{(iv)}$	84	0.25	1.125	315 ± 5	482.9	546.9	64.0
GCZS5316$^{(v)}$	130	0.312	1.046	338 ± 2	468.0	545.6	77.6
GCHC10152$^{(vi)}$	119	0.350	1.073	330 ± 5	508.4	602.0	93.6
GCHC10146$^{(vii)}$	156.4	0.402	1.093	324 ± 5	492.0	601.0	109
NLC1791$^{(viii)}$	109	0.452	1.073	330 ± 5	518.4	631.4	113

$^{(i-vii)}$ from Qingdao Grand Winton International Co. Ltd., China; $^{(viii)}$ from Military Univ. of Technology, Poland.

#### Dispersion of refractive index

Figure 4 shows the effect of the refractive index dispersion on the transmittance of a selected cholesteric sample. We compare two numerically calculated transmittance spectra $T(\lambda )$ in an ${8}\,\upmu \hbox {m}$ thick CLC cell with pitch length $p={0.300}\,\upmu \hbox {m}$. In one case, we have assumed that the refractive indices vary with wavelength according to some typical exemplary (5CB nematic liquid crystal) dispersion relations, $n_o(\lambda )$ and $n_e(\lambda )$ obtained from^46^ and given in the caption of Fig. 4. To obtain the second spectrum, we have assumed that the refractive indices are independent of wavelength and equal to the values of these functions at $0.500\,\upmu\hbox{m}$. It turns out that when taking into account the selected dispersion relations, the photonic band gap of the material becomes significantly narrower—for 9% (from ${59}\,\hbox {nm}$ to ${54}\,\hbox {nm}$) for this particular material—than in the calculation where the wavelength dependence of refractive indices is neglected. The reason for this is that the ordinary refractive index, corresponding to the band edge at the smaller wavelength is effectively larger at the lower band edge wavelength, compared to the value at $0.500\,\upmu \hbox{m}$. Contrary, the extraordinary refractive index, corresponding to the band edge at the larger wavelength is effectively smaller at the upper band edge wavelength, compared to the value at $0.500\,\upmu\hbox{m}$. Therefore, the band edges are moved closer together. In other calculations presented in this paper, we neglect the dispersion relations for simplicity.

#### Role of birefringence and angle between polarisation and director at incident plane on the transmittance

**Figure 5:**
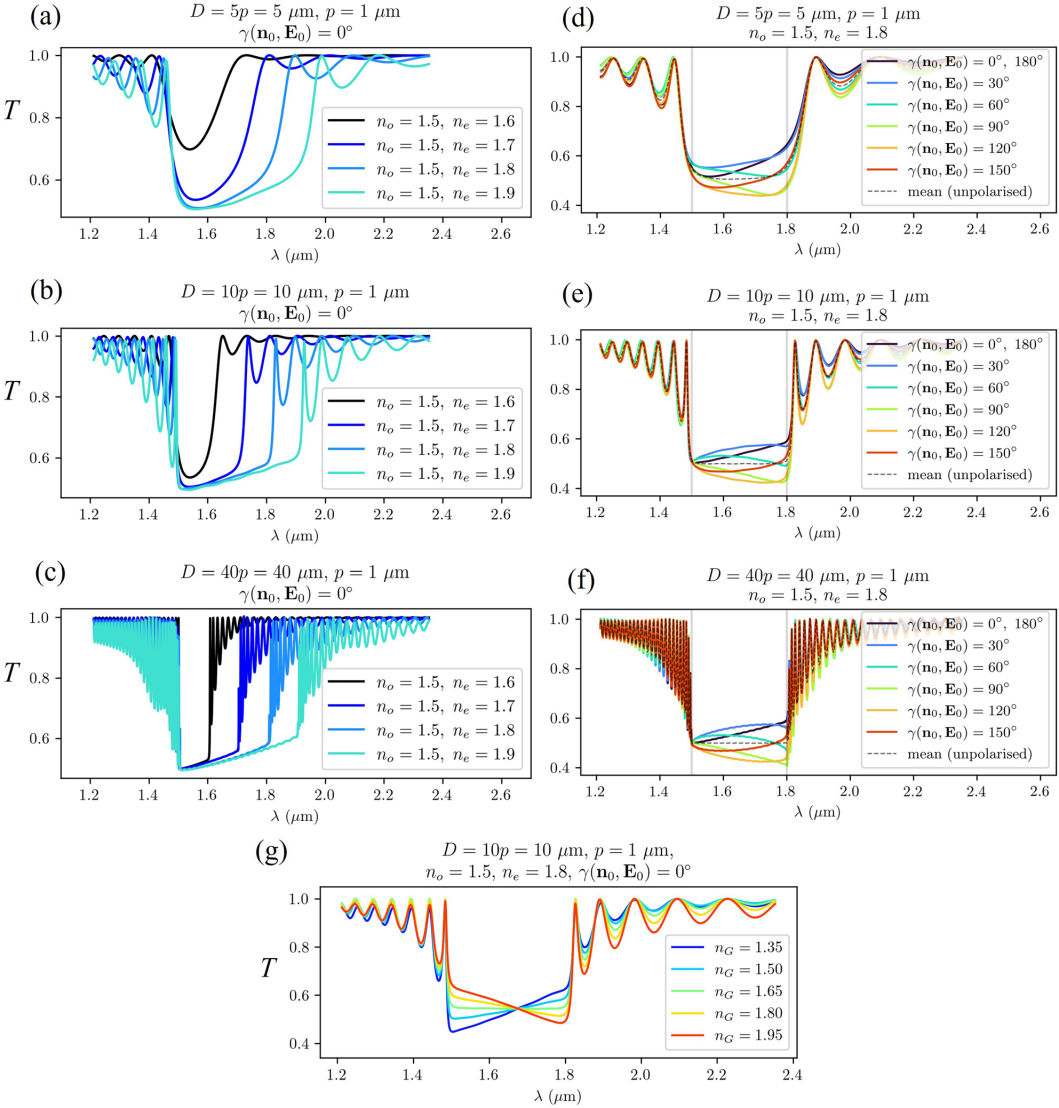
(**a,b,c**) Simulated transmittance spectra of CLC cells for different combinations of ordinary and extraordinary refractive indices in differently thick cells for linearly polarised light with polarisation parallel to the director at the incident plane $\gamma ({\textbf{n}}_o,{\textbf{E}}_0)=0^\circ$ in *D*/*p* is 5, 10, and 40 pitch thick cells. (**d,e,f**) Transmittance spectra of CLC samples with fixed refractive indices $n_o=1.5$, $n_e=1.8$ for different angles between the electric field’s polarisation and the director at the incident plane $\gamma ({\textbf{n}}_0,{\textbf{E}}_0)$ in *D*/*p* is 5, 10, and 40 pitch thick cells. The dashed lines represent the spectra of unpolarised light. (**g**) Transmittance spectra of the CLC with refractive indices $n_o=1.5$, $n_e=1.8$ and thickness $D=10p={10}\,\upmu \hbox {m}$ at different refractive indices $n_G$ of the confining isotropic material (glass). This is the only result in which we varied $n_G$. In all the others, we assumed its value to default at 1.5.

Figure 5 shows the calculated transmittance spectra for different sample thicknesses *D*, refractive indices $n_o$, $n_e$, and angles between the incident linear polarisation of the electric field and the director field at the edge of the sample $\gamma ({\textbf{n}}_o, {\textbf{E}}_0)$. As shown in panel (a), not only a sufficient number of cholesteric pitches but also a sufficiently large birefringence $\Delta n$ is required to reflect half of the linealry polarised incident light with wavelengths within the band gap. In sufficiently thick samples (Fig. 5b, c), the refractive indices determine only the position and the width of the band gap. Panels (d-f) in Fig. 5 show the dependence of the spectrum on the angle $\gamma ({\textbf{n}}_o,{\textbf{E}}_0)$. Transmittance is practically unaffected by this angle at wavelength $\lambda _1=n_op$ and at the peaks of the spectrum, but it can change with $\gamma ({\textbf{n}}_0,{\textbf{E}}_0)$ at wavelengths between $\lambda _1=n_o p$ and $\lambda _2=n_ep$. In particular, on the bandedge at $\lambda _2=n_ep$, the transmittance is $T(n_ep)\approx 0.6$ if $\gamma ({\textbf{n}}_0,{\textbf{E}}_0)=0^\circ$, while at $\gamma ({\textbf{n}}_0, {\textbf{E}}_0)=90^\circ$, it is considerably smaller, $T(n_ep)\approx 0.4$. This effect occurs due to refractive index mismatch between $n_G$ and $n_e$. If $n_G$ does not match $n_o$ either, similar behaviour is observed at the other band edge too. The effect of the refractive index of the isotropic material confining the CLC ($n_G$) on the transmission of linearly polarised light with $\gamma ({\textbf{n}}_0, {\textbf{E}}_0)=0^\circ$ is shown in panel (g) of Fig. 5. The slope of the spectral curve within the band gap changes from increasing to decreasing as $n_G$ increases from 1.35 to 1.95. The opposite trend is observed for linearly polarised light with $\gamma ({\textbf{n}}_0, {\textbf{E}}_0)=90^\circ$. In both cases, the spectral curve is horizontal within the bandgap when $n_G=(n_o+n_e)/2=1.65$. If we calculate the spectra $T(\lambda )$ for different angles $\gamma ({\textbf{n}}_0, {\textbf{E}}_0)$, and take their mean value at each wavelength, we obtain the transmission spectrum of unpolarised light. These spectra are indicated by dashed lines in panels (d,e,f) of Fig. 5. It turns out that the transmission within the bandgap is constant $T\approx 0.5$. This is true for different refractive indices $n_G$.

#### Transmittance spectra of divergent beams

The simulated transmittance spectra are noticeably different in shape from those measured experimentally (Fig. 3). In the simulations, we assume that the plane waves pulses are incident on the cholesteric liquid crystal sample, while in experiments, a non-coherent light source is focused on the sample. This allows waves to propagate through the sample not only along the helical axis but also at an angle.

**Figure 6:**
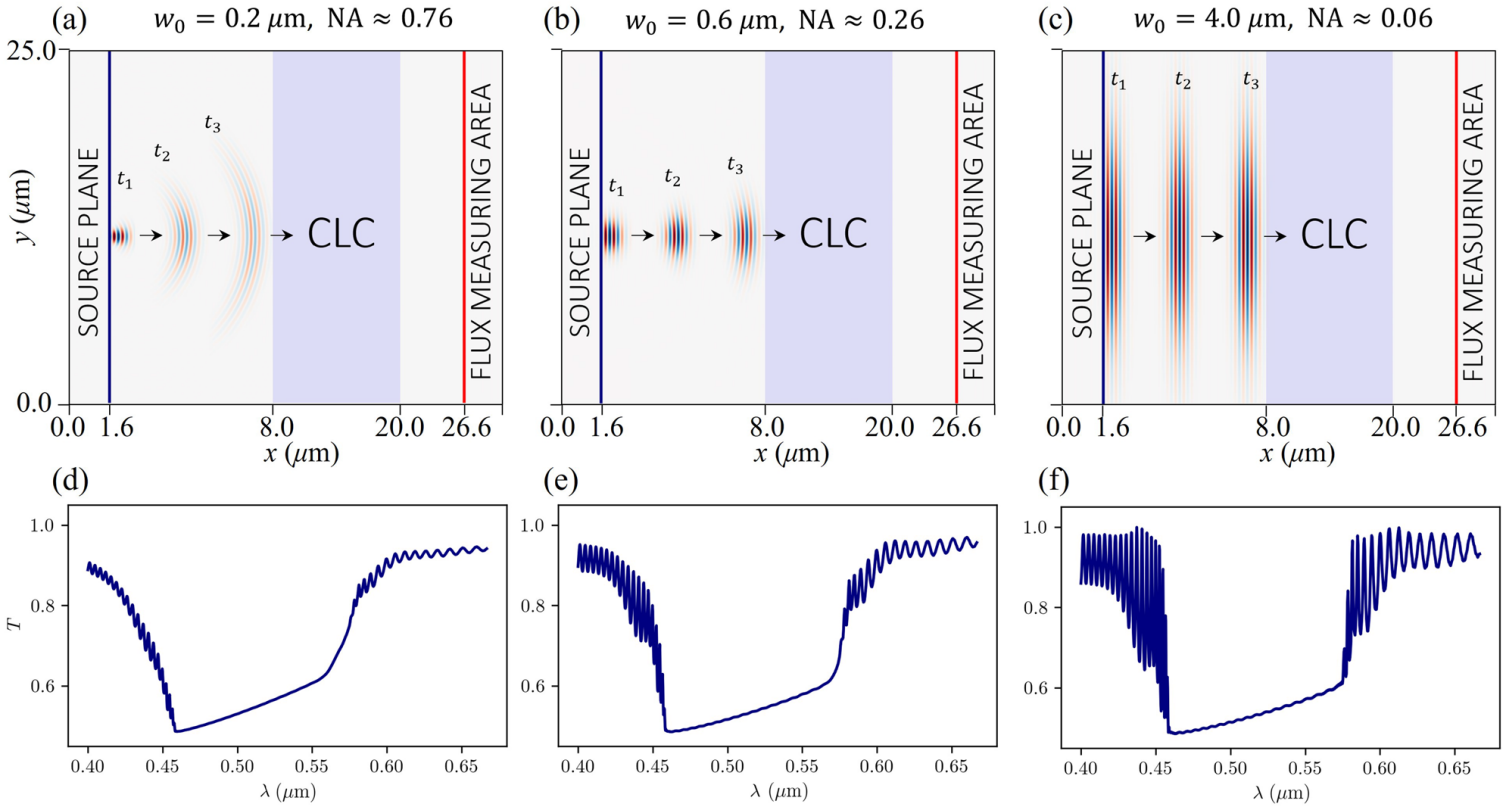
(**a,b,c**) Schematics of three different simulation setups. (**d,e,f**) Corresponding transmittance spectra $T(\lambda )$ of Gaussian beams with linear polarisation ($\gamma ({\textbf{n}}_0,\textbf{E}_0)=0^\circ$) and different waist thicknesses $w_0$. Smaller waist thicknesses correspond to higher numerical aperture NA and, thereby, smoother transmittance spectra. Parameters $p={0.300}\,\upmu \hbox {m}$, $n_o=1.525$, $n_e=1.909$, $D={12}\,\upmu \hbox {m}$ were used in these simulations. Beam focus is chosen to be at the source plane, which is placed ${6.4}\,\upmu \hbox {m}$ away from the CLC sample. Note that schematics in (**a,b,c**) are not to scale.

To include these effects in numerical simulations, we study the transmittance of a Gaussian beam pulses with different beam divergences $\theta =\overline{\lambda _0}/\pi n_G w_0$ given by the waist width $w_o$, the central vacuum wavelength of the pulse $\overline{\lambda _0}$ and the refractive index of glass $n_G$. Figure 6 shows that larger beam divergence (and thus larger numerical aperture $\textrm{NA}=n_G\sin (\theta )$)) causes the oscillations in the spectra outside the band gap to vanish gradually. This could be explained by the shift of the transmittance spectrum when the direction of light propagation through the CLC sample is not parallel to the helical axis^6^. In our case, the spectrum is actually the sum of the transmittance spectra for different directions, which causes the oscillations outside the band gap to be averaged out.

#### Role of incident polarisation on the transmittance

The transmittance of light through cholesteric samples is strongly affected by the polarisation of the incoming light. Half of the linearly polarised light, which can be considered as the sum of two opposite circular polarisations, is reflected, and half is transmitted. The same is true for unpolarised light. Circularly polarised light with the same handedness as the structure (in this case RCP) is almost completely reflected for wavelengths between $n_op$ and $n_ep$, while circularly polarised light with the opposite handedness (LCP) is almost completely transmitted.

**Figure 7:**
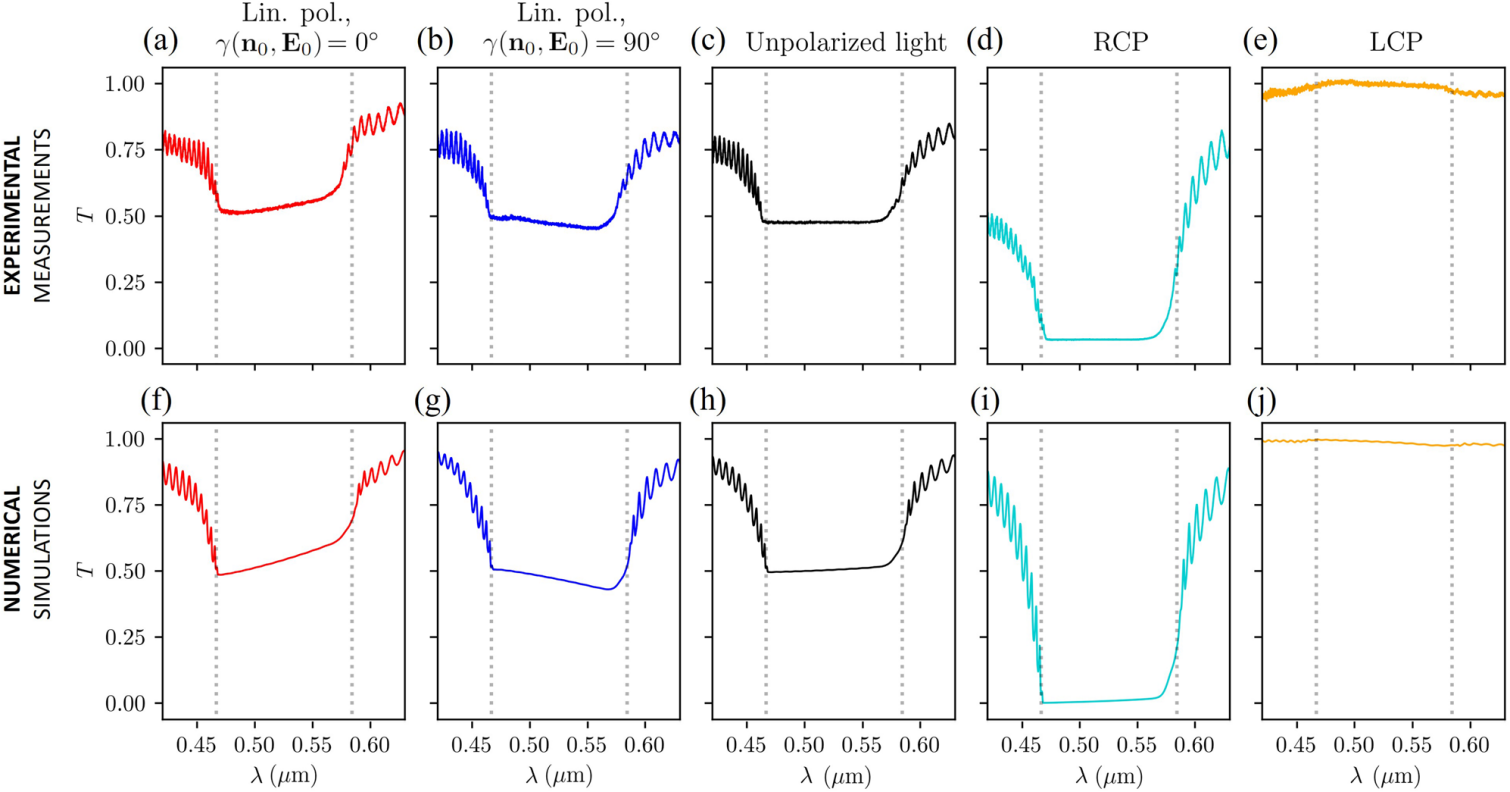
(**a**–**e**) Experimentally measured transmittance spectra of GCHC10146 cholesteric liquid crystal sample with right-handed helix for different polarisations: linear parallel (**a**), linear perpendicular (**b**), unpolarised light (**c**), right-handed circular polarisation (RCP) (**d**), and left-handed circular polarisation (RCP) (**e**) The experimentally measured transmission spectra also include the reflections at the air-glass interface. (**f**–**j**) Numerically calculated transmittance spectra of a Gaussian beam pulse with waist size $w_0=0.35\,\upmu \text {m}$ and different electric field polarisation combinations: linear parallel (**f**), linear perpendicular (**g**), unpolarised light (**h**), RCP (**i**), and LCP (**j**). Parameters used: $D={8}\,\upmu \hbox {m}$, $n_o=1.525$, $n_e=1.909$, $p={0.306}\,\upmu \hbox {m}$. In numerical simulations, reflections on the glass-air interface are ignored as the source is located inside the glass.

For liquid crystal GCHC10146 doped with R-5011 chiral dopant in a $D= {8}\,\upmu \hbox {m}$-thick cell with refractive indices $n_o = 1.525$, $n_e = 1.909$ (measured in a racemic mixture of R and S dopant), transmittance spectra for different polarisations of light were measured: for linear polarisation (parallel and perpendicular to the director field at the cell boundary), unpolarised light, and both circular polarisations, as shown in Fig. 7. Corresponding numerical calculations were performed, where we fitted the pitch length and the divergence of the incoming Gaussian beam to match the band gaps. The spectrum of unpolarised light was calculated as the average of the spectra of different linear polarisations for angles $\gamma ({\textbf{n}}_o, {\textbf{E}}_0)=0^\circ ,\ 10^\circ , \ldots ,\ 350^\circ$. Indeed, a very good qualitative agreement is observed for all polarisations.

### One-dimensional photonic eigenmodes in finite-length CLC resonators

**Figure 8:**
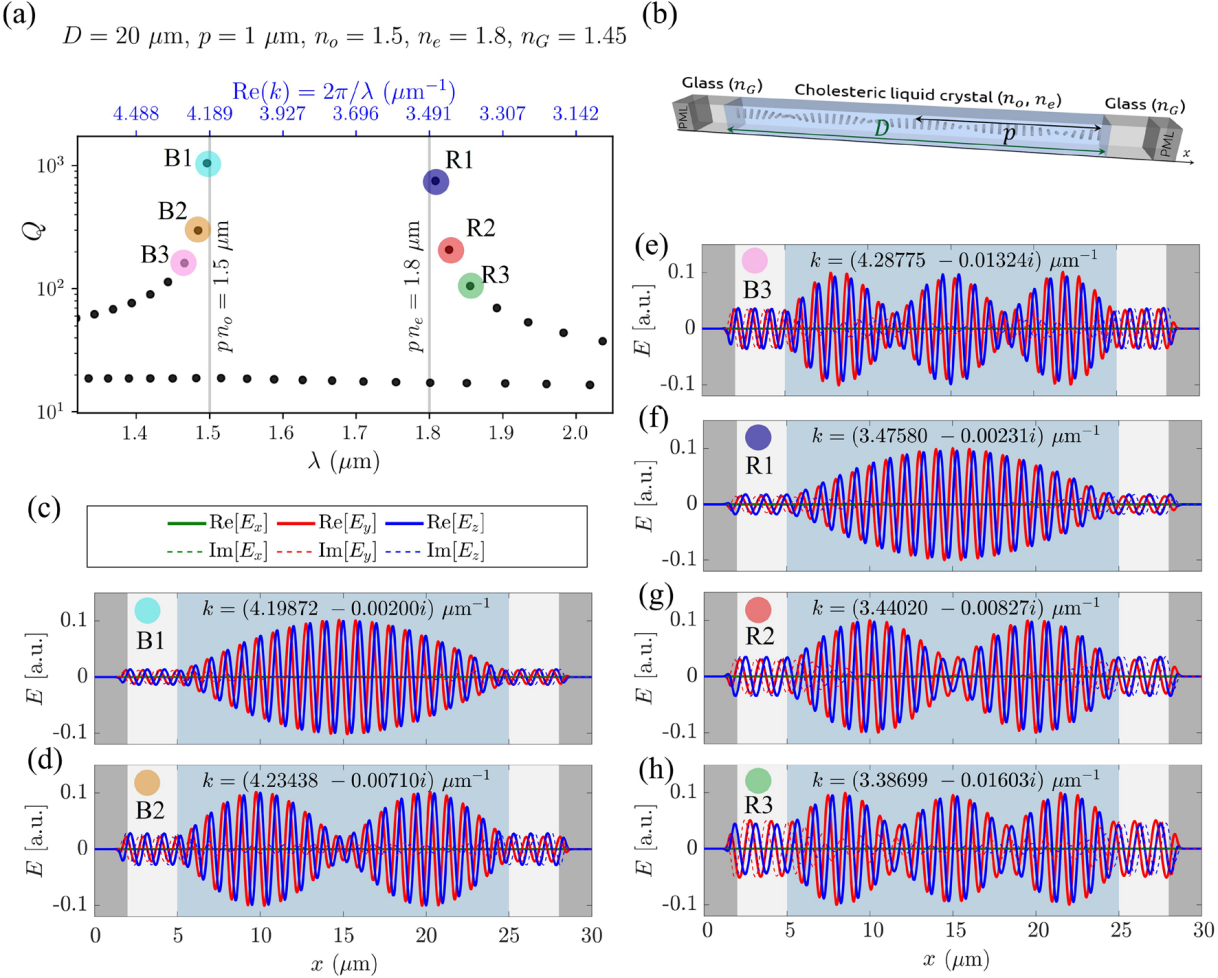
(**a**) *Q*-factors of the eigenmodes in the region of the band gap $p\; n_o<\lambda <p\; n_e$ (**b**) Schematic of the simulation domain geometry. (**c**–**h**) Electric field profiles of selected blue (B) and red (R) photonic eigenmodes in the vicinity of the band gap.

**Figure 9:**
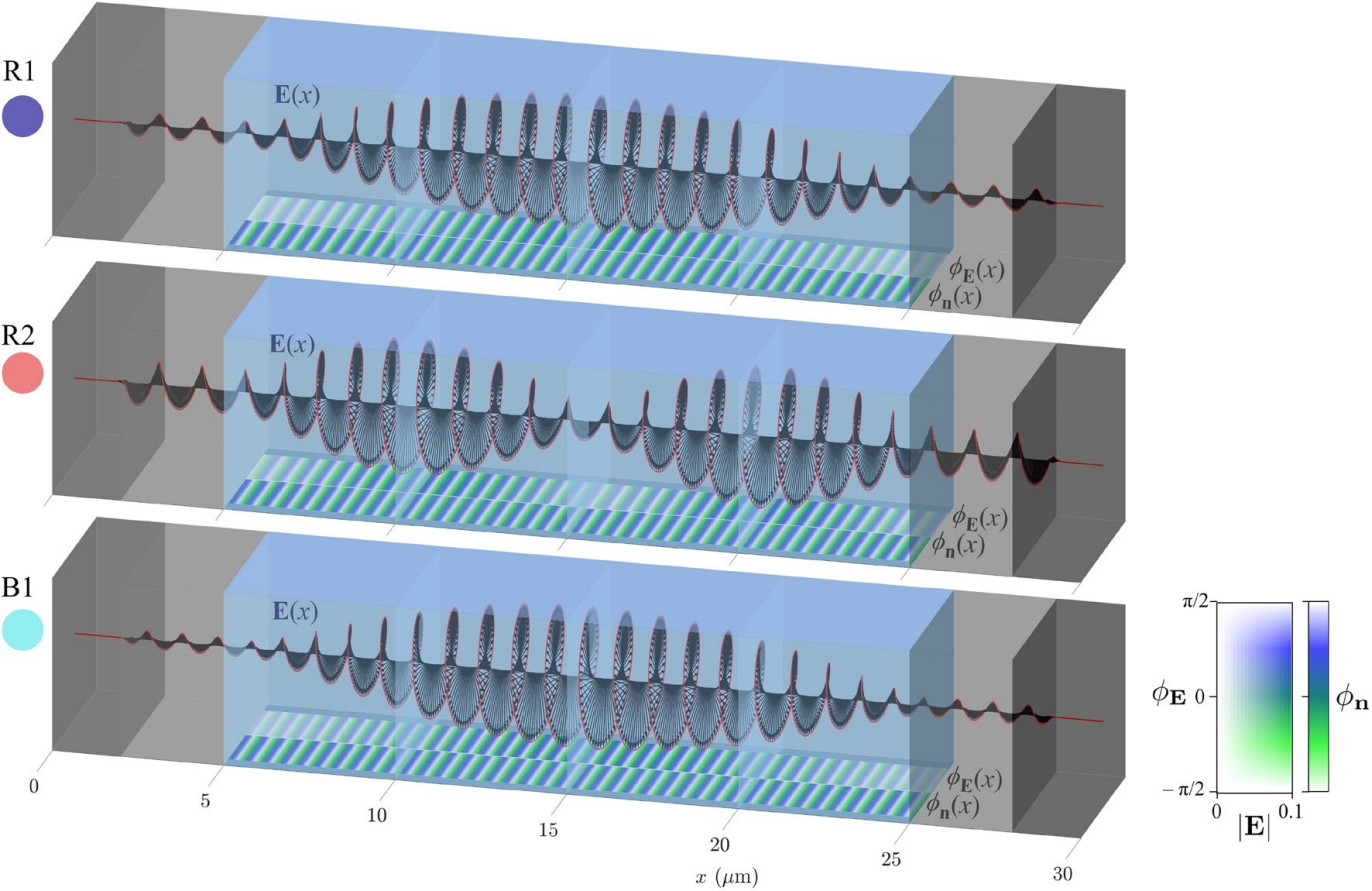
Three-dimensional representation of electric field vectors ${\textbf{E}}(x)$ of selected eigenmodes R1, R2, and B1 from Fig. 8. At the bottom surfaces, the orientation of the director $\phi _{\textbf{n}}=\arctan (n_z/n_y)$ and the orientation of the electric field $\phi _{\textbf{E}}=\arctan (E_z/E_y)$ inside the cell are shown in colours for comparison. Both red and blue modes have the same handedness as the CLC helix. In red modes, ${\textbf{E}}$ and ${\textbf{n}}$ are in phase in the areas of high electric field amplitude, whereas in blue modes, they are shifted by $90^{\circ }$. Relative angle between the electric field and the director $\gamma =|\phi _{\textbf{E}}-\phi _{\textbf{n}}|$ is shown in Fig. 11.

Cholesteric liquid crystals can also perform as photonic resonators for light of different frequencies, as determined by different photonic eigenmodes. We calculated these eigenfrequencies and eigenmodes using the FDFD method (as explained in Methods). As for transmittance, we consider a cholesteric liquid crystal sample with thickness *D*, pitch length *p*, and refractive indices $n_o$, $n_e$ confined between two glass plates with refractive index $n_G=1.45$, as shown in Figs. 1 and 8b. The thickness of glass plates $d_G$ is assumed to be $5\,\upmu\hbox{m}$, ending by $2\,\upmu\hbox{m}$ thick perfectly matched layers on both sides of the resonator. The calculated electric field profiles of resonant photonic eigenmodes of the cholesteric sample in the frequency region of the photonic band gap with corresponding eigenfrequencies and *Q*-factors are shown in Fig. 8. These modes can be classified into two distinct branches—blue (B modes: B1, B2,...on the blue side of the band gap), and red (R modes: R1, R2,...on the red side of the band gap). Modes B and R are all right-circularly polarised (same handedness as the structure) and exist only outside the photonic band gap. Eigenmodes with frequencies closest to the bandgap have the largest *Q*-factors. Selected modes B1, B2, and R1 are also represented in 3D images of the electric field profiles in Fig. 9, where the orientation of the electric field and the orientation of the liquid crystal director are compared. Distinctly, in our cholesteric system, there also exist numerical eigensolutions of Maxwell’s equations, which are left-circularly polarised (LCP) and emerge at all possible frequencies, also within the band gap, as shown in the spectrum in Fig. 8a. However, these modes have significantly smaller *Q*-factors, which is expected as LCP light of any frequency is fully transmitted through a right-handed CLC and therefore cannot show resonant behaviour. Also, changing the glass thickness $d_G$ in the numerical simulation causes a change in the spectrum of these modes, whereas the spectrum of B and R modes remains unchanged. In turn, we conclude that these left-handed modes are physically less relevant in the context of resonators, and we only focus on the resonant B and R modes in the rest of this article.

**Figure 10:**
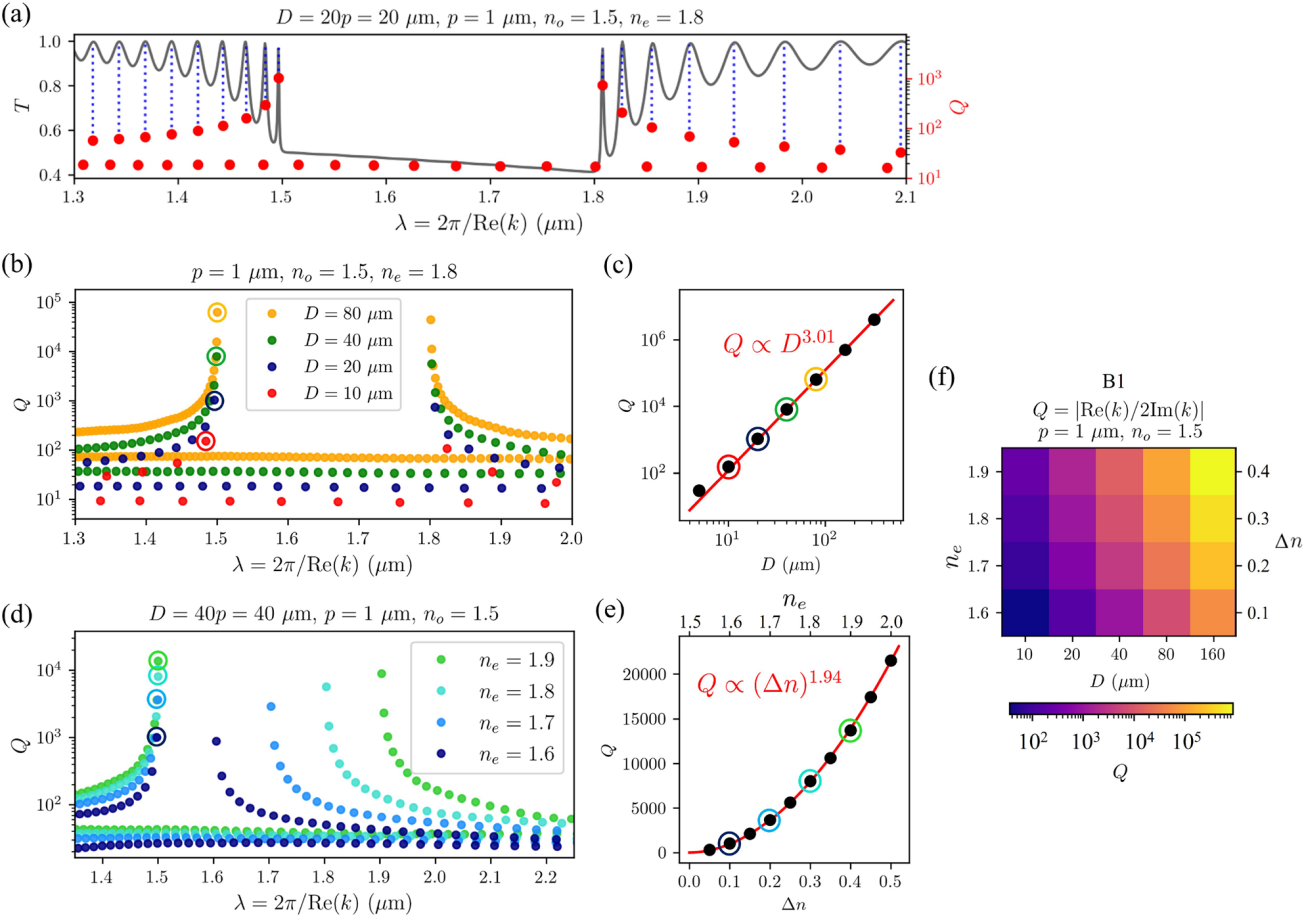
(**a**) Overlap between the peaks in the transmittance spectrum and the frequencies of the eigenmodes with the same handedness as the director. (**b**) *Q*-factors for different thicknesses of the sample. (**c**) *Q*-factor of the B1 mode in differently thick cells. For this example, it can be seen that the *Q*-factor as a function of the thickness *D* increases approximately as $Q\propto D^{3.01}$. (**d**) *Q*-factors for different birefringences (different extraordinary refractive indices $n_e$ at constant ordinary refractive index $n_o=1.5$) and constant thickness $D={40}\,\upmu \,\hbox {m}$. (**e**) *Q*-factor of the B1 mode for different birefringences of the sample at constant thickness. The *Q*-factor of this particular mode increases with birefringence approximately as $Q\propto (\Delta n)^{1.94}$. (**f**) The combined effect of birefringence $\Delta n$ and cell thickness *D* on the *Q*-factor of the B1 mode.

The eigenfrequencies of the modes that exist in a CLC resonator are closely related to the transmittance spectra. Figure 10a shows that the peaks in the $T(\lambda )$ spectrum (where $T=1$) correspond to the frequencies of the eigenmodes B and R which means that the electromagnetic plane waves with frequencies corresponding to the eigenfrequencies of the eigenmodes are completely transmitted through the cholesteric liquid crystal, even though the light is trapped within the sample due to internal reflections. Plane waves with frequencies somewhere between the eigenfrequencies are partially reflected, as shown in the transmission spectra.

Figure 10b shows that in thicker resonators with a larger number of pitches, there are more eigenmodes within a certain frequency range. Thus, the differences between adjacent eigenfrequencies are smaller. In the context of laser design, this can be important because a certain emission spectrum of a dye in a thicker CLC sample thus effectively overlaps with a larger number of the resonator’s eigenfrequencies. It is also clear that in thicker cells, the *Q*-factors of all modes are larger. Figure 10c shows the dependence of *Q*-factors for B1 modes as a function of cell thickness, i.e. the number of cholesteric layers. The eigenfrequencies of the eigenmodes are also affected by the refractive indices and the birefringence $\Delta n$, as shown in Figure 10d and e. Larger birefringence results in a wider band gap but it also means that the gradient of the effective refractive index for a given polarisation is larger, and hence the reflectivity is also larger. This leads to more light being trapped within the resonator, which corresponds to a higher Q factor. The combined influence of thickness and birefringence on the *Q*-factor of the mode B1 is shown in Fig. 10f.

**Figure 11:**
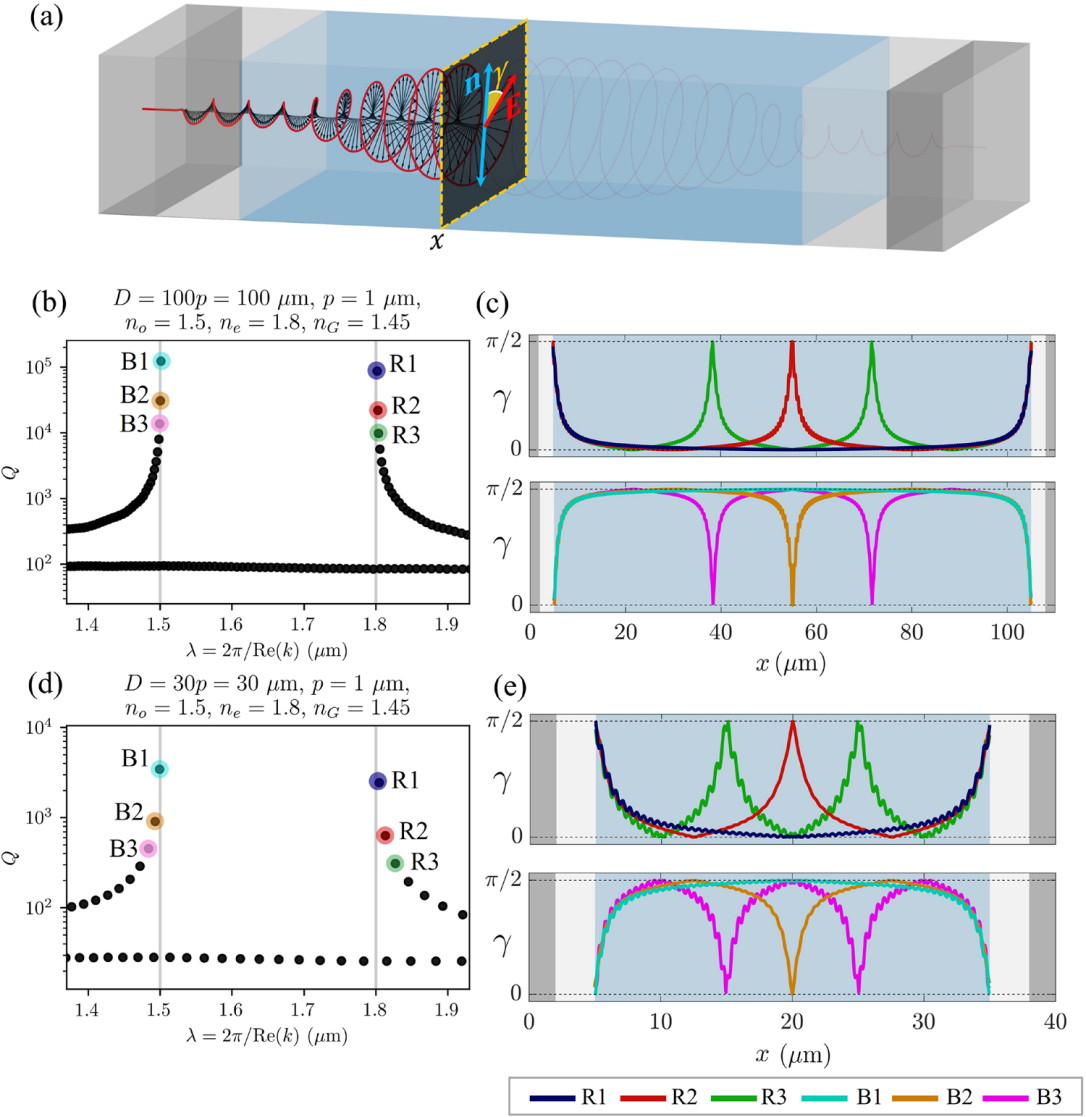
*Q*-factors of the eigenmodes in ${100}\,\upmu \hbox {m}$- (**b**), and ${30}\,\upmu \hbox {m}$-thick (**d**) CLC cells and corresponding spatial dependence of the relative angles $\gamma =\arccos {[(\textbf{n}\cdot {\textbf{E}})/|{\textbf{E}}|]}$ between the electric field polarisation ${\textbf{E}}$ and the director ${\textbf{n}}$ (as shown in (**a**)) for selected eigenmodes (**c,e**). The electric field of the mode R1 is perpendicular to the director at the boundaries whereas it is mostly parallel within the cell. The electric field of higher modes (R2, R3,...) is perpendicular to the director also in the nodes of their amplitude envelopes and it is parallel at maxima of amplitude envelopes. Opposite holds for the B modes.

In Fig. 8, the eigenmodes B1 and R1 look very similar, as do the B2 and R2, B3 and R3, etc. However, careful observation reveals that in a 20 pitches thick cholesteric sample, in mode R1, the electric field ${\textbf{E}}$ rotates from edge to edge by an angle of $19.5\cdot (2\pi )$, and in mode B1 by an angle of $20.5\cdot (2\pi )$. Actually, one can generalize this observation: In a $N_p$ pitches thick sample, the electric field of the mode R1 rotates by an angle of $2\pi (N_p-1/2)$, and that of mode B1 by an angle of $2\pi (N_p+1/2)$. Furthermore, it can be observed that for the R mode group, the electric field of the mode R2 rotates by $2\pi (N_p-1)$, of mode R3 by $2\pi (N_p-3/2)$,..., whereas for B modes, the electric field of mode B2 rotates by $2\pi (N_p+1)$, of mode B3 by $2\pi (N_p+3/2)$, etc. Evidently, the electric field of eigenmodes is, in general, neither parallel nor orthogonal to the liquid crystal director, as shown in Figs. 9 and 11. Nevertheless, the profile of the electric field in each mode adapts to the liquid crystal director such that the electric field is either parallel or perpendicular over the largest possible portion of the cell. Indeed, note that the distances at which the electric field rotates by an angle of $2\pi$ are not constant throughout the cell. Figure 9 also shows the comparison between the orientations of the electric field ${\textbf{E}}$ and the director field ${\textbf{n}}$ in colours, $\phi _{{\textbf{E}}}$ and $\phi _{{\textbf{n}}}$, respectively, whereas in Fig. 11, the spatial dependence of the relative angle $\gamma (x)=|\phi _{\textbf{E}}-\phi _{{\textbf{n}}}|$ between the electric field and the director is shown for selected modes B1, B2, B3, and R1, R2, R3. If the distances at which the electric field rotates by a full angle $2\pi$ were constant throughout the cell, panels (c) and (e) of Fig. 11 would show linear dependence, but this is not the case. For the R1 eigenmode, it turns out that the electric field is perpendicular to the director at both cell boundaries, whereas in between, it is parallel over most of the cell. Just the opposite is true for the mode B1. In modes R2, R3,...the field is perpendicular to the director at the cell boundaries, and in the nodes of the field envelopes, but in between, it is more or less aligned with the director. Again, the opposite holds for modes B2, B3,..., as shown in panels (c) and (e) in Fig. 11. Finally, effectively, the modes experience different effective refractive index profile as determined by the matching alignment between the electric field and the director, and higher modes see more effective regions of miss-alignment. Overall, this coupling results in different number of envelope (amplitude) peaks.

Understanding the eigenmodes of the passive resonator without gain is crucial for designing lasers. The *Q*-factor of the selected mode is essentially the ratio between the energy stored in the cavity and energy that is dissipated into the surroundings. Therefore the modes with higher *Q*-factor are more likely to lase or in other words, are expected to have lower lasing thresholds. The light of such modes is retained in the resonator for a longer time which allows more pumped atoms to be stimulated and emit light. We predict the shape of the mode inside the cavity. Later is important when we want to determine how to effectively pump the laser and efficiently use the gain. Additionally, LCs allow for the orientation of dissolved dye molecules^44,45^ and understanding the angle between the director field and electric field of the mode could enable engineering of lasers that emit different light modes. To understand the role of gain, further investigation by using methods that can effectively simulate lasing is needed.

## Discussion

In this work, we have analyzed the light transmittance through cholesteric liquid crystals and the corresponding photonic eigenmodes as conditioned by multiple system parameters and effects: (i) finite length of the cholesteric helical pattern, (ii) different material birefringence, (iii) different types of incoming polarisation, and (iv) relative angle between incoming polarisation and director. Experimental measurements of transmittance spectra have been systematically performed for several materials with different birefringences and with different polarisations of the incident light. Numerically, we calculated transmittance spectra for a general range of typical values of refractive indices, cell thicknesses, and incident polarisations. Additionally, we have analyzed the effect of the refractive indices dispersion on the transmittance spectra and shown how the transmittance spectra change when pulses with curved wavefronts propagate through the CLC cell. We have shown that the peaks in the transmittance spectra coincide with the eigenfrequencies of the CLC resonator eigenmodes and presented the geometry of these modes as well as their *Q*-factors. Overall, this work explores and outlines the properties of cholesteric liquid crystal helical patterns as photonic resonators and highlights their properties that are important for the design of liquid crystal micro-lasers and other soft-matter-based photonic devices.

## Methods

### Numerical method

In numerical calculations, we assume that the system is infinite along *y* and *z* axes, allowing us to perform simulations on a one-dimensional mesh with three-dimensional electric and magnetic field vectors, ${\textbf{E}}(x)=\left( E_x(x),E_y(x),E_z(x)\right)$ and ${\textbf{H}}(x)=\left( H_x(x),H_y(x),H_z(x)\right)$, respectively. Open boundary conditions are assumed at the boundaries of the system to prevent any additional reflections, which is modelled by adding a few wavelengths thick (${2}\,\upmu \hbox {m}$ in our simulations) perfectly matched layers (PMLs)^47,48^ that absorb electromagnetic fields at the boundaries of the simulation domain, as also shown in Fig. 1.

Transmittance spectra of the CLC samples of different thicknesses are calculated using the finite-difference time-domain (FDTD) method^49^, using Meep software^50^ which solves Maxwell’s equations in the time domain. The transmittance spectrum $T(\lambda )$ is calculated as a ratio between the spectrum of a pulse with Gaussian time-dependence transmitted through a CLC sample and a spectrum of an equal pulse transmitted through the isotropic glass. By time-limiting the pulse, we ensure that the spectrum of the pulse is spectrally broad and covers the frequency range of interest. In principle, we could choose any other smooth function with similar time dependence instead of a Gaussian profile. Here, a plane-wave source with time-dependence proportional to $\exp (-i\omega t-(t-t_0)^2/2w^2)$ and arbitrary polarisation is placed at $x=d_G/2$ though and has infinite size along *y* and *z* axes. The area at which the transmitted flux spectra are measured is placed at the same distance from the sample as the source but on the other side of the sample, at $x=d_G+D+d_G/2$. The central spectral frequency of the source $\omega$ and the spectral width 1/*w* are set to $\omega =1/(p\overline{n})$, where $\overline{n} =(n_o+n_e)/2$ and $1/w=\omega$ so that the spectrum roughly overlaps with the range of frequencies in which reflection of light is observed. In calculations, we take actual experimental values (when making comparisons, typically approx. ${300}\,\hbox {nm}$) or a generic value of $p={1}\,\upmu \hbox {m}$. The simulations are performed until the mean amplitudes of the electromagnetic fields at the flux measuring area decay by a factor of $5\cdot 10^{-5}$ compared to the maximum ones during each run. Numerical simulations were performed for different combinations of material parameters *D*, $n_o$, $n_e$ and angles between linear light polarisation and anchoring direction at the incident surface $\gamma ({\textbf{n}}_0, {\textbf{E}}_0)$, shown in Fig. 1.

The electromagnetic field in resonators can be described as a superposition of photonic modes as ${\textbf{E}} ({\textbf{r}},t)=\sum _\mu \Psi _\mu ({\textbf{r}}) e^{-ik_\mu t}$, where $\Psi _\mu ({\textbf{r}})$ are the given modes. We calculate the modes as eigensolutions of Maxwell’s equations using the finite-difference frequency-domain method by solving the eigenproblem^32,51^:

2 $$\begin{aligned} \nabla \times \nabla \times \Psi _\mu ({\textbf{r}})-k_\mu ^2 \underline{\varepsilon ({\textbf{r}})}\Psi _\mu ({\textbf{r}}) = 0, \end{aligned}$$

where each eigenmode $\Psi _\mu ({\textbf{r}})$ has its corresponding eigenvalue $k_\mu =\omega _\mu / c_0$, sometimes also referred to as an eigenfrequency when $c_0=1$ is assumed. The dielectric tensor $\underline{\varepsilon ({\textbf{r}})}$, which describes the liquid crystal structure and confining glass, is taken as real (i.e. without absorption) except in the PML region, where it has an imaginary component to assure absorption and imitate open boundary conditions. Consequently, both the calculated electric field profile of each resonator mode $\Psi _\mu ({\textbf{r}})$ and the eigenfrequency $k_\mu$ are always complex. The decay rate of the modes, is inversely proportional to the quality factor (*Q*-factor), defined as^52^:

3 $$\begin{aligned} Q_\mu =\left| \frac{\text {Re}[k_\mu ]}{2\text {Im}[k_\mu ]}\right| . \end{aligned}$$

Especially, we focused on frequencies in the vicinity of the photonic band gap, as these modes are most interesting for possible laser design.

### Experimental methods and materials

Experimentally, we studied a series of different nematic materials with different birefringence ranging from 0.07 to 0.45. The CLCs investigated in this study were prepared by mixing pure nematic liquid crystals with the right-handed chiral dopant R-5011 (Grandin Chem Co Ltd, China) above the clearing temperature of pure NLCs. The helical twisting power (HTP) of R-5011 is around ${\sim }116\, \upmu \text {m}^{-1}$ at $20^\circ$C, and the amount of chiral dopant was adjusted to obtain nearly the same pitch in all CLC mixtures at room temperature where the experiments were performed. The birefringence and the clearing temperature of pure nematic liquid crystals are listed in Table 1.

To study the pitch length and the photonic band gap, as prepared CLCs were introduced into planar-aligned wedge cells by capillary action in the isotropic phase. The wedge cells were made of two 0.5 mm thick glass plates (Soda-lime glass, AGC Flat Glass (Thailand) Public Company Limited) that were covered by 20–30 nm thin layer of polyimide (Nissan SE-5291). The polyimide (PI) was rubbed prior to cell assembling, and the rubbing directions on each glass plate were set antiparallel to prevent any splay due to the surface pre-tilt on the PI. The thickness of wedge cells was from $1\,\upmu\hbox{m}$ to $10\,\upmu\hbox{m}$, and the same type of cells was used in all experiments. The pitch lengths of different CLCs were measured by using the conventional Grandjean-Cano wedge method^53^.

We used a Nikon polarising microscope (ECLIPSE TE2000-U) equipped with an unpolarised white light source (Halogen lamp 12V, 100W) and condenser to measure transmission or reflection spectra of CLC samples. The light, transmitted through the CLC sample was collected using a low numerical aperture 20$\times$ objective (Nikon, Plan Fluor 20$\times$/0.5) and sent to a spectrophotometer with 0.5 nm resolution (Andor, Shamrock SR-500i), equipped with cooled EM-CCD camera (Andor, Newton DU 970N). The photonic band gap was measured for unpolarised white light at a sample thickness of ${8}\,\upmu \hbox {m}$ for all CLCs. The transmittance data was collected for 1 s exposure time with a spectrometer slit size of ${25}\,\upmu \hbox {m}$.

## Data Availability

Data supporting this study’s findings are available upon reasonable request from the first author J.Z, and will be archived in Zenodo Community of the ERC project *Light-operated logic circuits from photonic soft-matter (LOGOS)*.
